# Maleylated-BSA-Coated Gold Nanopopcorns for Pulmonary Delivery of Quercetin

**DOI:** 10.3390/ijms27198808

**Published:** 2026-10-01

**Authors:** Onkar Nigade, Bala Prabhakar, Francesco Trotta, Pravin Shende

**Affiliations:** 1 Shobhaben Pratapbhai Patel School of Pharmacy and Technology Management, SVKM’s NMIMS, Vile Parle (W), Mumbai 400056, India; 2 Department of Chemistry, University of Turin, Via Pietro Giuria 7, 10125 Torino, Italy; 3 National Institute of Pharmaceutical Education and Research (NIPER), S.A.S. Nagar (Mohali) 160062, India

**Keywords:** nanopopcorns, gold, tuberculosis, sustained release

## Abstract

A non-motile bacterium, *Mycobacterium tuberculosis* (Mtb), causes tuberculosis (TB), an infection of the lower respiratory tract. Currently, rifampicin, isoniazid, ethambutol, and pyrazinamide are used as first-line treatment for drug-susceptible TB for two months, followed by four months of rifampicin and isoniazid. This treatment plan is linked to serious hepatic, dermatological, and hematopoietic adverse effects. The exploration of natural substances like quercetin for treating TB was driven by the significant side effects of the standard therapy protocol. Protein-coated gold nanopopcorns were developed as a dry-powder inhaler to deliver tailored medication to the lungs. The objective of this research article was to assess the effectiveness of quercetin-loaded gold nanopopcorns against Mtb H37Ra cells. The gold nanopopcorns prepared using the Turkevich method and subsequently coated with maleylated BSA (m-BSA) showed an adsorption efficiency of 87.02 ± 2.82%, particle size of 153.2 ± 26.45 nm, and a zeta potential of 29.3 ± 7.80 mV. In vitro release studies showed rapid initial release within 2 h, with a total drug release of 79.85 ± 1.33% over 24 h, with inhibition of 56.36% of H37Ra cells (IC_50_ = 2 μg/mL). These findings provide preliminary evidence for the potential of PNPs as a pulmonary quercetin delivery platform, warranting further evaluation in macrophage-based and tuberculosis disease models.

## 1. Introduction

Tuberculosis is a disease of the lower respiratory tract, caused by *Mycobacterium tuberculosis* (Mtb) via zoonotic and anthropozoonotic transmission. Mtb is a non-motile, non-sporulated, rod-shaped, and acid-fast-staining aerobic bacterium possessing a lipid-rich cell wall. Mtb spreads through airborne particles exhaled by an infected person, after being trapped by protective mucous membranes. It comes into contact with macrophages suspended in the airway and is phagocytosed, where it multiplies [1]. As per WHO guidelines, current therapy for drug-susceptible tuberculosis includes 2 months of orally administered ISD, RIF, PYR, and ETH followed by 4 months of ISD and RIF [2]. However, the treatment is associated with several side effects such as skin rash, hepatitis, thrombocytopenia, vestibulocochlear nerve damage, kidney damage, optic neuritis, etc. [3]. To overcome such problems, phytoconstituents like chalcones, alkaloids, terpenes, phenols, and flavonoids are currently under investigation as potential antitubercular agents [4]. Quercetin, a flavanol compound, shows activity against Mtb by inhibiting glutamine synthetase, an enzyme responsible for producing L-glutamine, an essential component of the Mtb cell wall. Quercetin inhibits isocitrate lyase, an enzyme responsible for the persistence of Mtb in macrophages; the B subunit of DNA gyrase, β-KAS-3, which is an enzyme responsible for the formation of mycolic acid present in the mycomembrane; and uridine-5′-diphosphate galactopyranose mutase [5,6,7,8,9]. Quercetin showed 99.30 ± 0.26% inhibition of Mtb H37Rv cells at a concentration of 200 μg/mL [10,11]. However, quercetin is a BCS class-IV compound that exhibits poor oral bioavailability (<10%), a variable oral elimination half-life of 11–28 h driven by extensive metabolism, and rapid systemic clearance (IV half-life of 0.7–2.4 h).

In nanomaterial technology, small particle size, large surface area, and surface charge strongly influence cellular internalization of metal nanoparticles. Additionally, the ability of metal nanoparticles to produce reactive oxygen species (ROS) is responsible for imparting antibacterial activity. Nanoparticles of different metals, like gold, silver, zinc, copper, iron, titanium, etc., exhibit unique antibacterial properties, making them potential candidates for the treatment of infectious diseases [12,13]. Gold nanopopcorns are nanoparticles with irregular morphology, providing an inert and minimally invasive method for delivery of drugs like anti-inflammatory and anticancer agents. The rationale for selecting specialized nanomaterials is their high drug-loading potential and excellent protein conjugation capacity, which offer targeted action. The modified protein maleylated BSA (m-BSA), a ligand for scavenger receptor-B1, a receptor found on the surface of macrophages, was coated on top of drug-loaded nanopopcorns and investigated as a potential macrophage-targeting ligand owing to its reported interaction with scavenger receptors [14]. Despite the reported antimycobacterial potential of quercetin and the utility of gold-based nanostructures for drug delivery, the integration of quercetin-loaded gold nanopopcorns with a maleylated bovine serum albumin (m-BSA) surface coating for pulmonary delivery has not been adequately investigated. In the present study, we developed a quercetin-loaded gold nanopopcorn system functionalized with m-BSA and systematically evaluated its physicochemical characteristics, drug-release behavior, antimycobacterial activity against *Mycobacterium tuberculosis* H37Ra, and pulmonary drug deposition in rats. The study therefore establishes a formulation platform that combines the high surface area and irregular morphology of gold nanopopcorns with m-BSA surface functionalization for pulmonary delivery of quercetin. Accordingly, the objective of the present study was to develop and characterize m-BSA-coated, quercetin-loaded gold nanopopcorns and to investigate their in vitro antimycobacterial activity and pulmonary drug disposition following administration.

## 2. Results

### 2.1. PSZ, PDI, and ZEP

GNPs, QNPs, and PNPs were evaluated for PSZ and ZEP, as depicted in Table 1. PSZ of GNPs was 34.42 ± 12.48 nm with a PDI of 0.457. QNPs exhibited a PSZ of 125.9 ± 56.89 nm and a PDI of 0.361. PNPs showed a further increase in PSZ (153.2 ± 26.45 nm). The ZEP of GNPs was +39 ± 10.98 mV. QNPs exhibited a slightly lower ZEP of 34.8 ± 5.66 mV. Moreover, there was a further reduction in ZEP (+29.3 ± 7.80 mV) in the case of PNPs.

### 2.2. % Adsorption Efficiency (% AEF)

AEF was performed in triplicate for QNPs and was found to be 87.02 ± 1.29%.

### 2.3. In Vitro Release Studies

**Figure 1:**
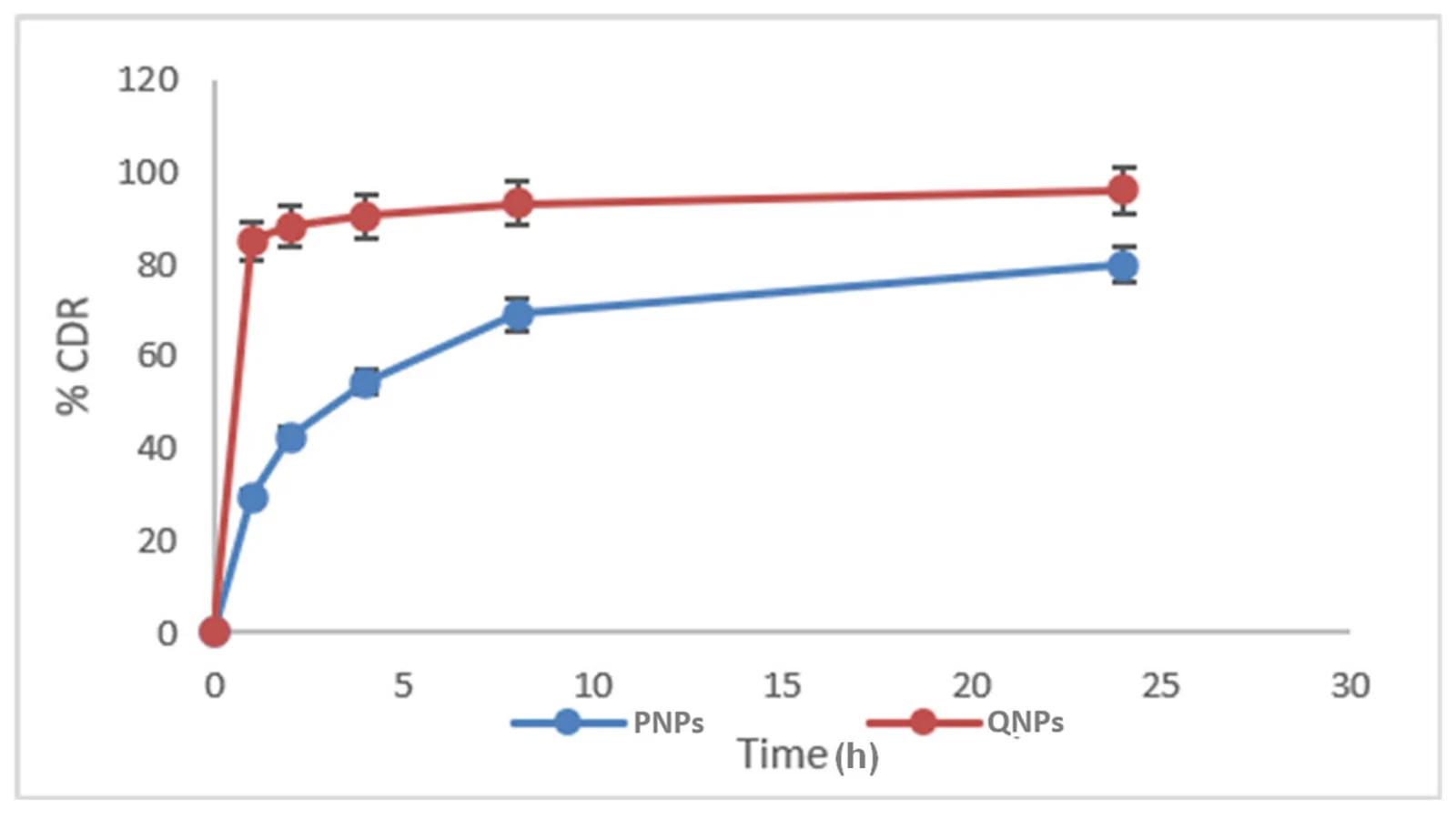
In vitro release of QNPs and PNPs.

The drug release studies for QNP and PNPs formulations were performed in triplicate and showed rapid release initially, followed by a sustained release pattern after 4 h, as shown in Figure 1. QNPs showed a cumulative drug release of 95.97 ± 4.67% in 24 h, with rapid drug release for up to 4 h, followed by sustained release through 24 h. PNPs showed a cumulative release of 79.85 ± 3.92% in 24 h, demonstrating rapid initial release, with 54.25 ± 2.25% of the drug released in the initial 4 h, followed by sustained release through 24 h.

### 2.4. FTIR Studies

**Figure 2:**
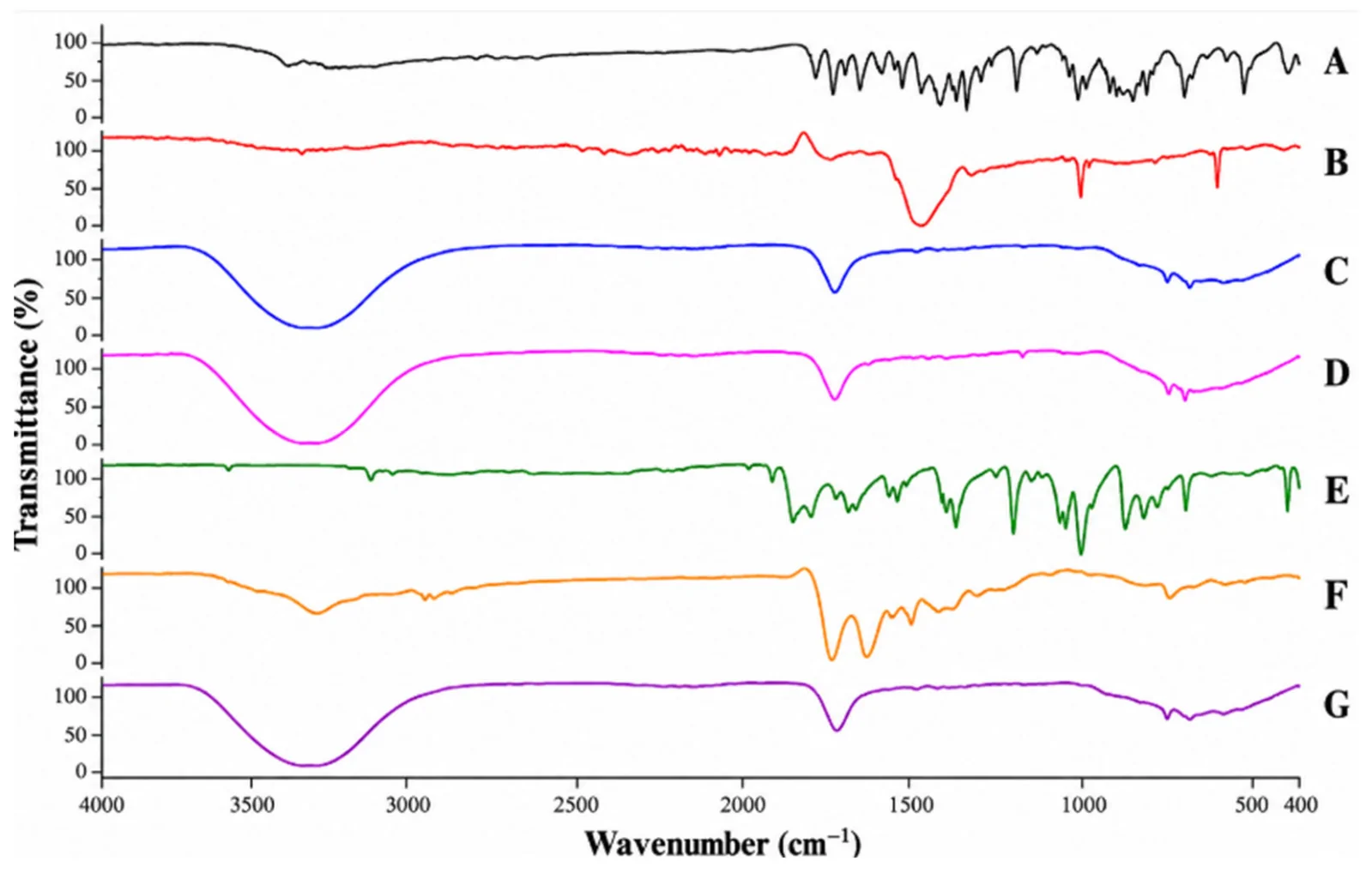
FTIR spectra of (**A**) quercetin, (**B**) HAuCl4, (**C**) GNPs, (**D**) QNPs, (**E**) maleic anhydride, (**F**) BSA, and (**G**) PNPs.

FTIR spectrum of quercetin in Figure 2 shows peaks at 3264.21 cm^−1^ and 1663.55 cm^−1^, confirming the presence of -OH and C=O groups, respectively. Moreover, the peaks at 1607.99 cm^−1^ and 1560.79 cm^−1^ represent symmetric and asymmetric C-C=C stretching, respectively. GNPs show peaks at 3337.95 cm^−1^, 1635.99 cm^−1^, and 596.64 cm^−1^, thereby confirming the existence of OH, C=O, and C-Cl groups, respectively. QNPs exhibit peaks at 3338.60 cm^−1^, 599.75 cm^−1^, 1638.50 cm^−1^, 1561.46 cm^−1^, and 1258.82 cm^−1^ for -OH, C-Cl, and C-C=C symmetric stretching, C-C=C asymmetric stretching, and C-O stretching, respectively. BSA shows peaks at 3326.24 cm^−1^, 1636.29 cm^−1^, and 606.84 cm^−1^, indicating the presence of 1° amine, an amide group, and a NH group. FTIR spectrum of maleic anhydride shows peaks at 1854.47 cm^−1^, 1775.22 cm^−1^, and 1056.06 cm^−1^, indicating the presence of C-C=O symmetric stretching, C-C=O asymmetric stretching, and C-O stretching, respectively. FTIR spectrum of PNPs shows peaks at 1062.46 cm^−1^ corresponding to the C-O stretching of maleic anhydride, 1647.81 cm^−1^ and 1392.74 cm^−1^ corresponding to the amide (I) helix and amide (III) helix of BSA, and 596.64 cm^−1^ corresponding to the C-Cl group of auric chloride.

### 2.5. DSC Studies

**Figure 3:**
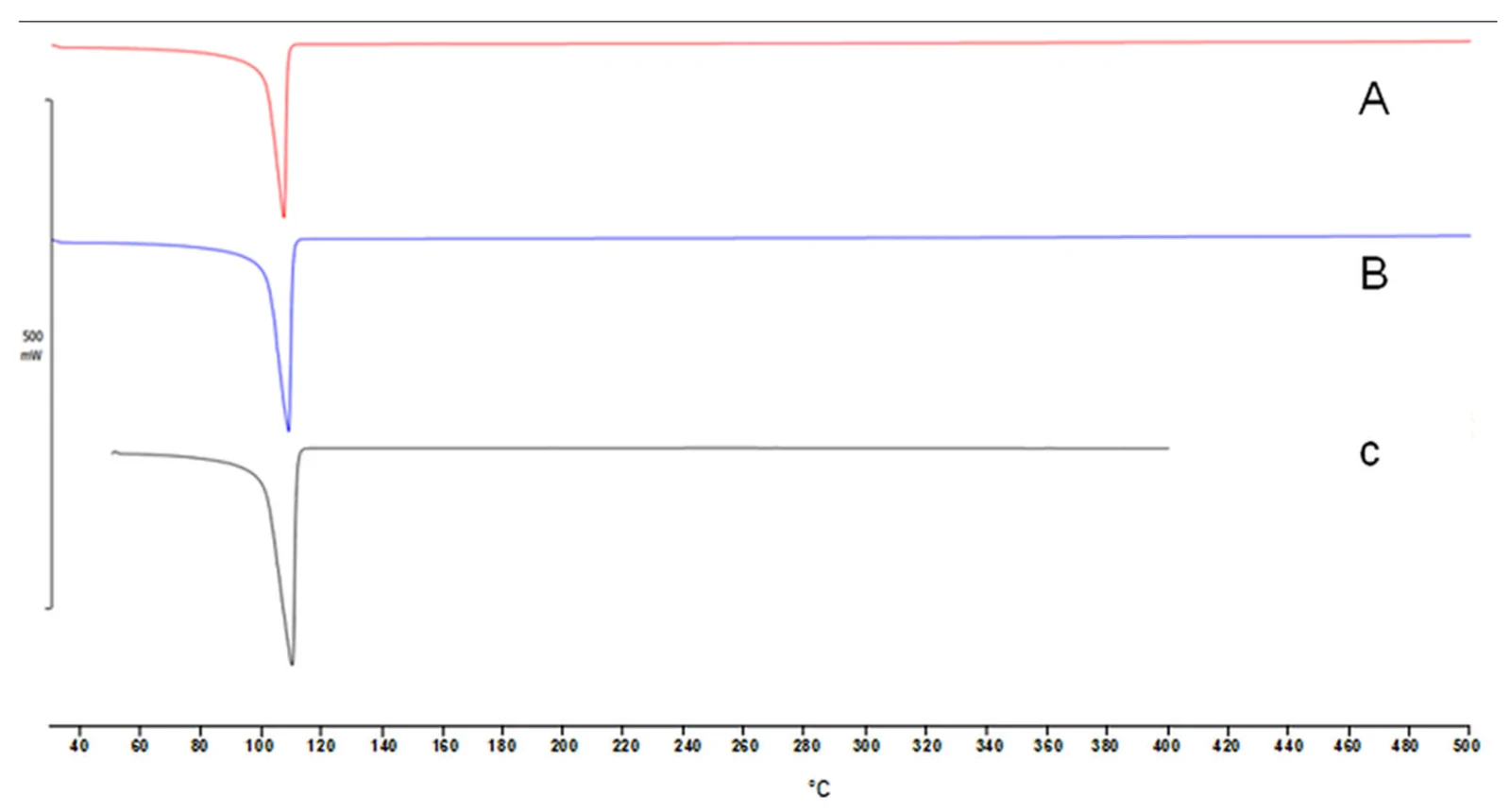
DSC thermograms of (**A**) GNPs, (**B**) QNPs, and (**C**) PNPs. The red, blue, and gray traces represent GNPs, QNPs, and PNPs, respectively.

To evaluate the solid-state characterization and phase transitions of the individual components and final formulations, DSC analyses were performed, and the results are shown in Figure 3. Pure gold chloride exhibited a characteristic thermal profile consisting of an exothermic peak followed by an endothermic peak at 251.74 °C. The thermogram of pure crystalline quercetin revealed two distinct peaks at 132.69 °C and a sharp, prominent endothermic melting peak at 322.11 °C. Conversely, pure BSA displayed a single broad endothermic event centered at 75.96 °C. As illustrated in Figure 3, the thermograms of GNPs, QNPs, and PNPs shifted significantly compared to their individual ingredients and exhibited sharp endothermic peaks at 112.65 °C, 114.76 °C, and 115.94 °C, respectively. The characteristic sharp crystalline melting peak of quercetin at 322.11 °C completely disappeared in both the QNPs and PNPs formulations.

### 2.6. Morphology

**Figure 4:**
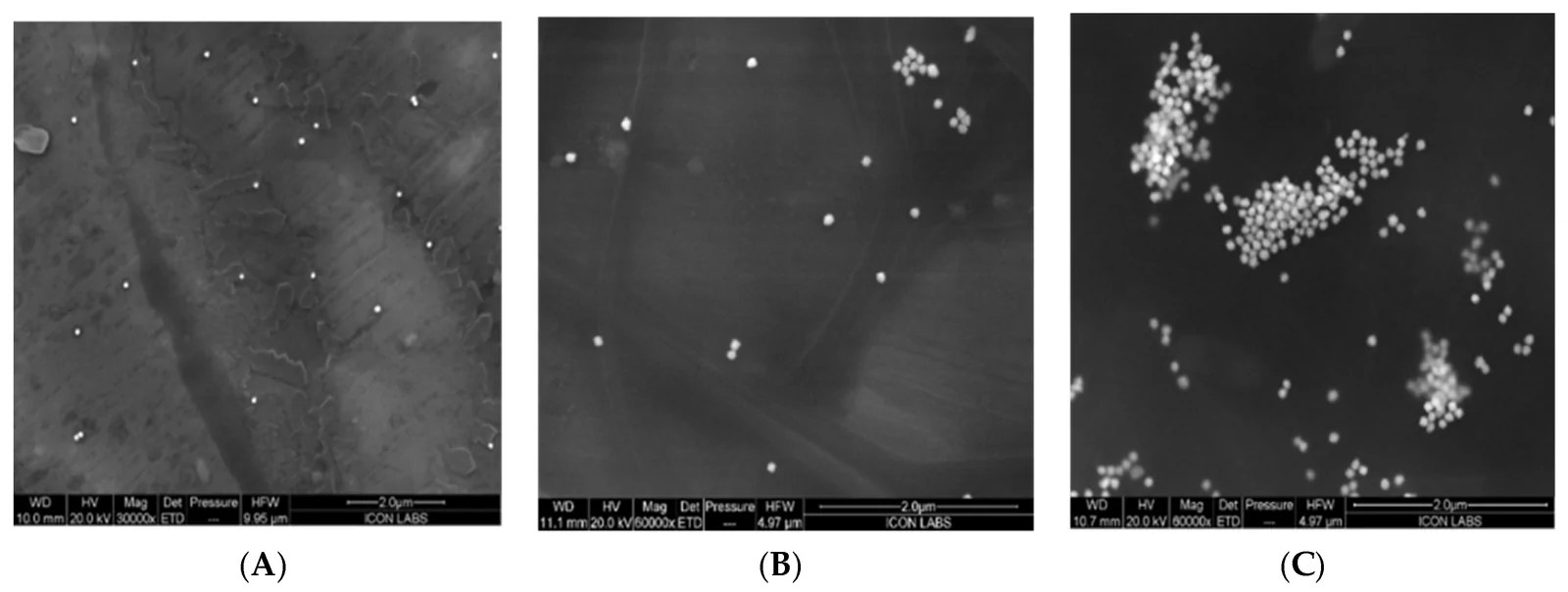
SEM images of (**A**) GNPs, (**B**) QNPs, (**C**) PNPs.

**Figure 5:**
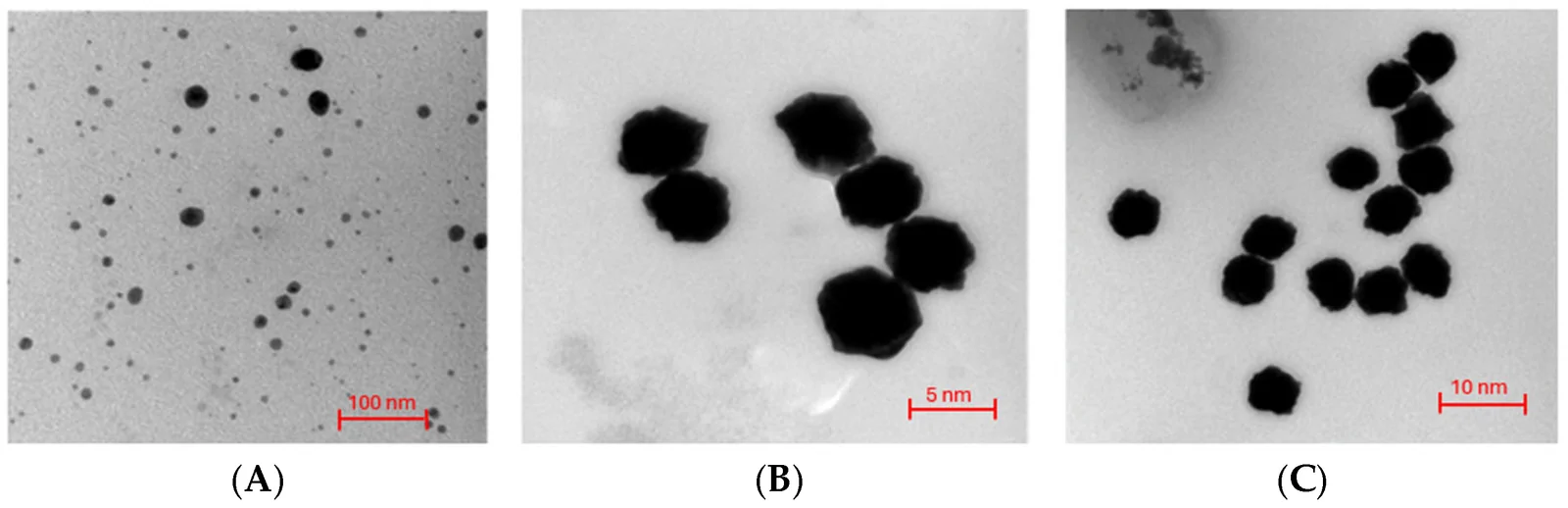
TEM images of (**A**) GNPs, (**B**) QNPs, and (**C**) PNPs.

SEM studies provided information about the morphology of the GNP, QNP, and PNPs formulations. The GNP formulation demonstrated a tiny particle size with a slightly irregular shape (Figure 4A), but adsorption of quercetin resulted in a slightly increased particle size in the case of the QNP formulation (Figure 4B). Moreover, protein-coated nanopopcorns (PNPs) exhibited an even larger size and an irregular shape (Figure 4C). TEM analysis revealed the irregular structure of the GNP formulation (Figure 5A), with an average particle size of 34.42 ± 12.48 nm. Adsorption of quercetin on the surface of the nanopopcorns resulted in an increased average particle size (125.9 ± 56.89 nm) (Figure 5B). Development of an m-BSA coating did not significantly alter the morphology but substantially increased the particle size to 153.2 ± 26.45 nm (Figure 5C).

### 2.7. In Vitro Antimycobacterial Activity

**Figure 6:**
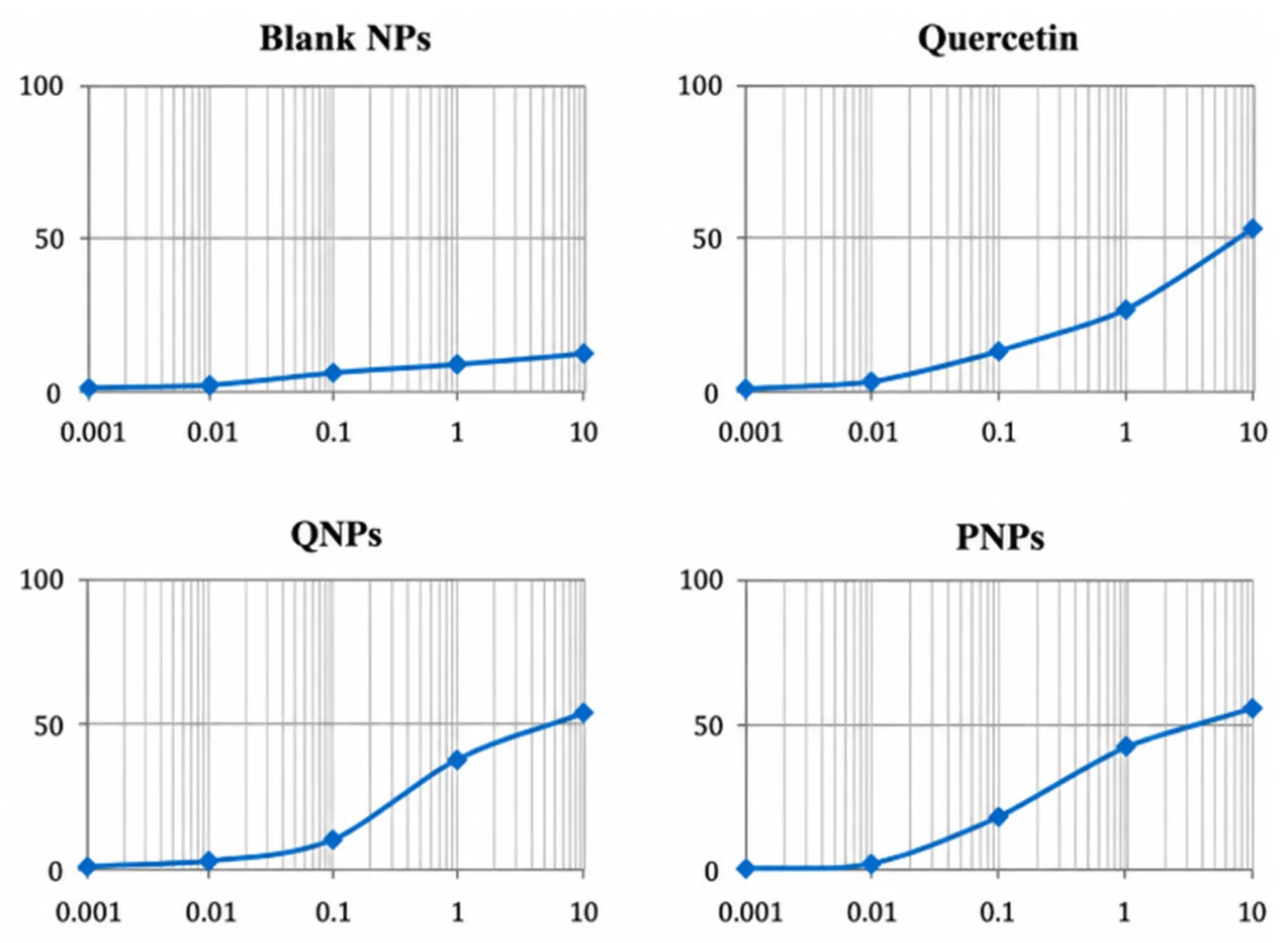
% Inhibition for Blank NPs, quercetin, QNPs, and PNPs.

The antimycobacterial activity of free quercetin, GNPs, QNPs, and PNPs was evaluated against the attenuated M. tuberculosis H37Ra strain using an extracellular culture-based assay as shown in Figure 6. The PNPs formulation exhibited the highest inhibitory activity of 56.36%, followed by QNPs, which showed inhibition of 53.23%. The quercetin solution showed similar effectiveness, inhibiting 52.34% of bacterial cells, whereas GNPs showed the lowest inhibition at 12.31%. The IC_50_ values observed for each formulation and % inhibition at different concentrations are summarized in Table 2. Gold nanopopcorns were ineffective in suppressing the growth of H37Ra cells but showed a consistent dose-dependent increase in % inhibition. Quercetin showed consistently greater inhibition of H37Ra cells at concentrations above 0.001 µM, and QNPs showed similar potency when compared to the quercetin solution. At concentrations above 0.1 µM, the PNPs formulation consistently showed greater inhibition than QNPs, GNPs, and quercetin. The inhibitory activity of PNPs below a concentration of 0.1 µM was lower than that of the other formulations.

### 2.8. Animal Studies

**Figure 7:**
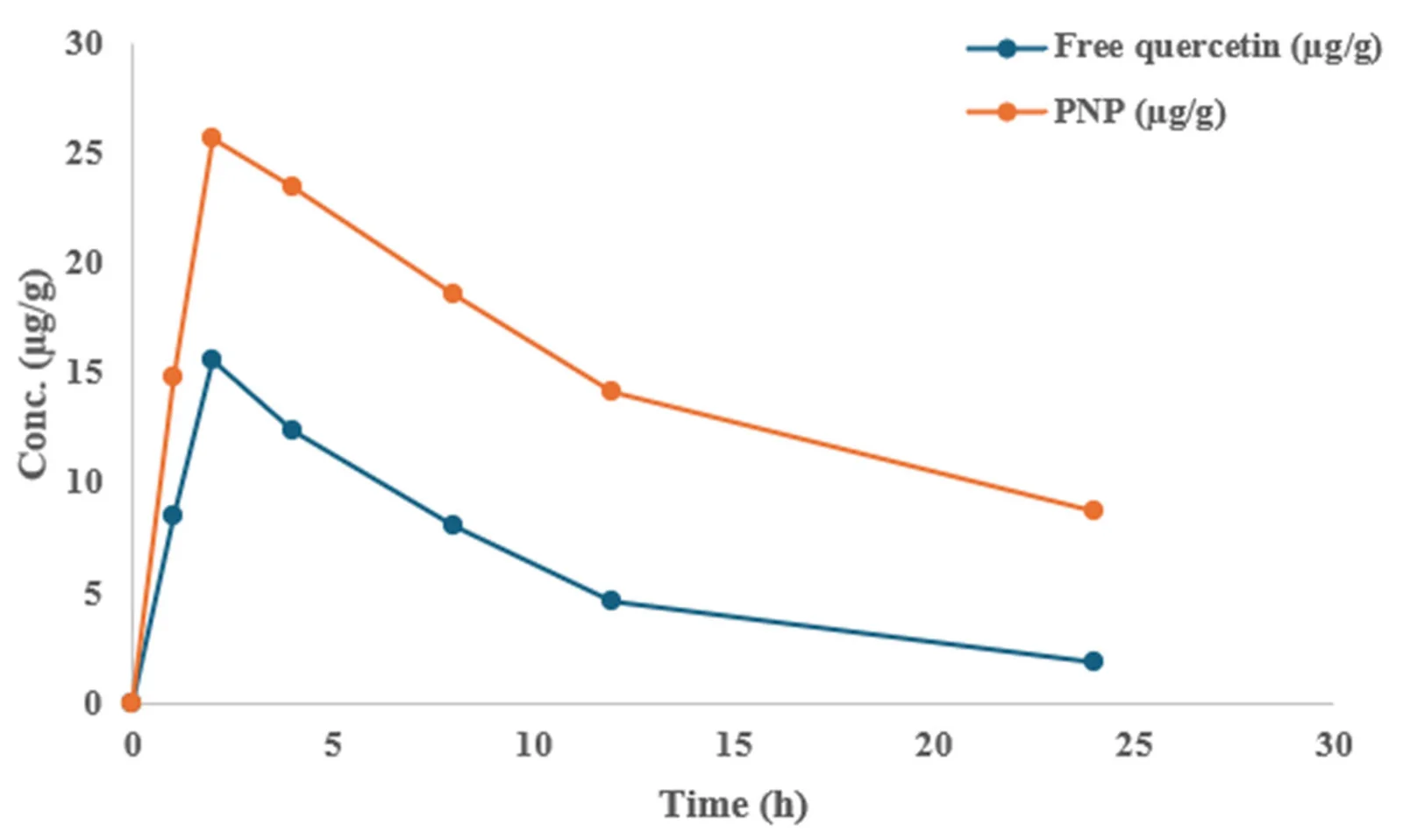
Pulmonary concentration - time profiles of quercetin following administration of free quercetin and PNPs in rats.

Quercetin was detected in lung tissue at all evaluated time points following administration of both free quercetin and PNPs (Figure 7). Free quercetin exhibited an increase in pulmonary concentration from 8.50 ± 1.20 µg/g at 1 h to a maximum concentration of 15.60 ± 2.10 µg/g at 2 h, followed by a progressive decline to 1.90 ± 0.40 µg/g at 24 h. In comparison, PNPs produced higher pulmonary quercetin concentrations, increasing from 14.80 ± 1.70 µg/g at 1 h to 25.70 ± 2.80 µg/g at 2 h, followed by a gradual decline to 8.70 ± 1.10 µg/g at 24 h (Table 3).

## 3. Discussion

### 3.1. PSZ, PDI, and ZEP

The PSZ of GNPs was mainly attributed to trisodium citrate acting as a strong reducing agent to reduce hydrogen tetrachloroaurate (Au^3+^)to Au0, which produced nanopopcorns with a high polydispersity index. NaBH_4_ acted as a strong nucleating agent and therefore produced monodisperse nanopopcorns. The increase in PSZ was attributed to adsorption of quercetin on the surface of nanopopcorns, and a further increase in PSZ was observed for PNPs, which was attributed to the protein coating of m-BSA on the surface of QNPs. The relatively high PDI values observed for GNPs, QNPs, and PNPs indicate a heterogeneous particle-size distribution that arises from the irregular morphology of the nanopopcorns, surface adsorption of quercetin, and subsequent m-BSA coating. The progressive increase in hydrodynamic diameter following drug loading and protein functionalization was accompanied by changes in the size distribution, reflecting the structural heterogeneity of the nanopopcorn system. The positive charge of GNPs was attributed to CTAB on the surface of nanopopcorns. QNPs exhibited a slightly lower ZEP due to the presence of hydroxyl groups of quercetin, which were adsorbed on the surface of nanopopcorns. Moreover, there was a further reduction in ZEP in the case of PNPs due to the presence of m-BSA.

### 3.2. % Adsorption Efficiency (% AEF)

A strong intermolecular interaction was observed between the quaternary ammonium head of CTAB and the hydroxyl group of quercetin, which contributed to the adsorption of the drug on the surface of nanopopcorns. The Na+ of sodium borohydride and trisodium citrate also contributed to the adsorption of quercetin on the surface of nanopopcorns owing to strong intermolecular interactions with the hydroxyl groups of quercetin. The irregular surface morphology and positive surface charge were also responsible for the formation of a protein corona around the nanopopcorn structures. The attachment of quercetin to gold nanopopcorns was achieved through surface adsorption rather than covalent conjugation. Quercetin contains multiple phenolic hydroxyl groups but lacks the free carboxyl group required for direct EDC/NHS-mediated coupling. Therefore, drug loading is attributed to non-covalent interactions, including hydrogen bonding, hydrophobic interactions, and adsorption onto the CTAB-functionalized nanoparticle surface. In contrast, EDC/NHS chemistry was employed during the subsequent surface functionalization step to facilitate immobilization of m-BSA onto the nanoparticle surface.

### 3.3. In Vitro Release Studies

The initial rapid release in the case of QNPs was attributed to the loosely bound drug, and the subsequent slow release of quercetin was due to the tightly bound drug because of its interaction with CTAB. Trisodium citrate and sodium borohydride, which acted as reducing, nucleating, and stabilizing agents, contributed to the sustained release of the drug. The drug release was comparatively lower and slower in the case of PNPs, which could be due to the binding of quercetin to m-BSA. The diffusion of the drug from the protein coating was responsible for the rapid initial release; the subsequent controlled release was due to the time required for quercetin to diffuse into the protein coating, followed by diffusion into the surrounding medium.

### 3.4. FTIR Studies

The peaks observed for QNPs indicated the adsorption of quercetin onto the surface of nanopopcorns. The peaks observed for PNPs indicated the successful formation of a protein corona around the nanopopcorns. The FTIR spectrum of QNPs demonstrated the presence of characteristic quercetin functional groups, supporting successful surface adsorption of quercetin onto the gold nanopopcorns. The FTIR data indicate drug loading but do not constitute evidence of covalent EDC/NHS-mediated conjugation.

### 3.5. DSC Studies

The broad endothermic event observed for BSA was characteristic of amorphous macromolecules undergoing dehydration and denaturation. The sharp, prominent endothermic melting peak of quercetin at 322.11 °C confirmed its highly crystalline nature. The absence of the drug’s melting endotherm indicated that quercetin was no longer present in its crystalline state. Instead, it converted into an amorphous form, molecularly dispersed within the polymeric matrix, or completely encapsulated/adsorbed onto the surface of the nanopopcorn structure.

### 3.6. Morphology

The increase in particle size following quercetin adsorption was attributed to the adsorption of quercetin onto the surface of nanopopcorns. The further increase in particle size of PNPs was associated with the development of the m-BSA coating. The use of EDC/NHS facilitated protein-coat formation on top of the drug-loaded nanopopcorns.

### 3.7. In Vitro Antimycobacterial Activity

The prominent inhibitory action of the QNP and PNPs formulations is likely due to rapid release of quercetin and subsequent inactivation of glutamine synthetase, isocitrate lyase, uridine 5′-diphosphategalactopyranosemutase, subunit B of topoisomerase II, and beta-KAS III [6,15]. The lower inhibitory activity of PNPs below a concentration of 0.1 µM may be attributed to lower drug release from the formulation, as shown in Figure 7. A limitation of the present study is that antimycobacterial activity was assessed using an extracellular H37Ra culture model rather than an intracellular macrophage infection model. Consequently, the enhanced activity observed with PNPs cannot be conclusively attributed to increased macrophage internalization or intracellular antimycobacterial efficacy. Moreover, macrophage uptake and scavenger-receptor-mediated internalization of PNPs were not experimentally quantified. Therefore, the maleylated-BSA coating should be considered a potential macrophage-targeting moiety, and its targeting efficacy requires further validation using cellular uptake and intracellular infection models. Recent evidence further emphasizes the importance of evaluating mycobacterial therapeutics within a host-cell context, as mycobacterial infection can modulate host-cell apoptosis, epithelial barrier integrity, and cellular signaling pathways [15,16]. Therefore, although the present extracellular H37Ra assay provides preliminary evidence of antimycobacterial activity, it does not recapitulate the complex host–pathogen interactions associated with intracellular infection. These findings support the need for future macrophage-based studies to determine whether PNP-mediated drug delivery translates into enhanced intracellular antimycobacterial activity.

### 3.8. Animal Studies

The pulmonary concentration–time profiles demonstrated distinct differences between free quercetin and the PNPs formulation. Free quercetin showed a rapid increase in pulmonary concentration followed by a pronounced decline, whereas PNPs maintained comparatively higher quercetin concentrations throughout the 24 h observation period. The higher C_max_ and AUC_0–24_ observed with PNPs indicate greater pulmonary exposure compared with free quercetin. Furthermore, the persistence of measurable quercetin concentrations at 24 h following PNPs administration suggests prolonged pulmonary retention of the drug. The slower terminal decline observed with PNPs was also reflected in the longer apparent half-life. These findings may be associated with the physicochemical characteristics of the nanopopcorn formulation and the sustained release behavior observed in the in vitro study. However, pulmonary tissue concentrations represent drug disposition within the lung and should not be interpreted directly as in vivo drug-release kinetics [14]. The observed pulmonary quercetin deposition following administration of PNPs may be associated with the physicochemical characteristics of the formulation and the sustained release of quercetin. The m-BSA coating may provide potential macrophage-targeting functionality based on its reported interaction with scavenger receptors; however, receptor-mediated uptake was not directly evaluated in the present study.

## 4. Materials and Methods

### 4.1. Materials

Silver(I) nitrate (AgNO_3_), auric chloride trihydrate (HAuCl_4_·3H_2_O), L-ascorbic acid (C_6_H_8_O_6_), bovine serum albumin (BSA), sodium borohydride (NaBH_4_), and trisodium citrate (Na_3_C_6_H_5_O_7_) were procured from Research-Lab Fine Chem Industries, Mumbai, Maharashtra, India. Cetyltrimethylammonium bromide (CTAB) was obtained from Loba Chemie Pvt. Ltd., Mumbai, Maharashtra, India. Ethyl(dimethylaminopropyl)carbodiimide (EDC), maleic anhydride, N-hydroxysuccinimide (NHS), and quercetin were purchased from Sigma-Aldrich Chemicals Pvt. Ltd. RAW264.7 mouse macrophage cells (Cat. No. CL-0190), TCMK-1 mouse renal tubular epithelial cells (Cat. No. CL-1260), HK-2 human renal proximal tubular epithelial cells (Cat. No. CL-0109), and THP-1 human monocytic cells (Cat. No. CL-0233) were obtained from Wuhan Pricella Biotechnology Co., Ltd., Wuhan, China.

### 4.2. Method

**Figure 8:**
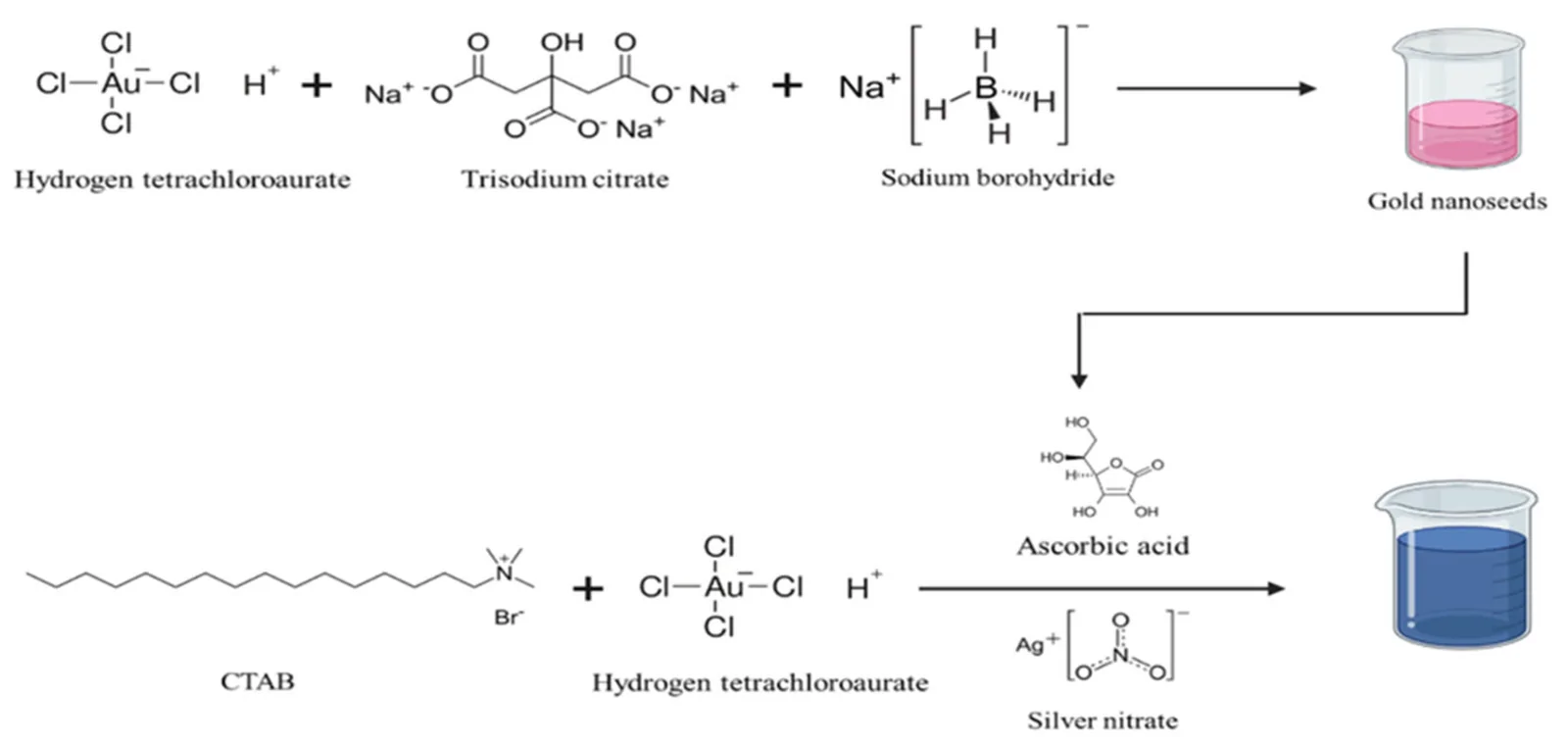
Preparation of gold nanoparticles.

Nanopopcorns were prepared according to the Turkevich method via a two-step seed-mediated growth process, as shown in Figure 8.

#### 4.2.1. Preparation of Spherical Nanoseeds (GNSs)

The first step involved the preparation of small, spherical gold nanoseeds (GNSs) using hydrogen tetrachloroaurate as the gold precursor, sodium borohydride as the strong reducing agent to trigger nucleation, and trisodium citrate as the stabilizing agent. Briefly, a solution of hydrogen tetrachloroaurate was prepared by adding 30 mg HAuCl_4_.3H_2_O in 2.5 mL of distilled water, and 0.5 mL of this solution was added to 18 mL of distilled water, followed by the addition of 60 μL trisodium citrate solution (20 mg in 1 mL) and 40 μL of NaBH_4_ (3 mg in 1 mL) under continuous stirring. The solution turned pink, indicating the formation of GNSs, and the prepared nanoseeds were refrigerated overnight at 4 °C.

#### 4.2.2. Preparation of Gold Nanopopcorns (GNPs)

The second step covered the preparation of gold nanopopcorns (GNPs) using CTAB (a shape-templating agent), L-ascorbic acid (a weak reducing agent), and previously prepared GNSs to produce nanoparticles with the desired morphology. Briefly, a sufficient quantity of CTAB (50.5 mg) was dissolved in distilled water (46.5 mL) by sonicating for 30 min. Then, auric chloride (2 mL) was added to this solution, which turned yellow after the addition of 500 μL of AgNO_3_ solution (8 mg in 5 mL). Subsequently, 1 mL of L-ascorbic acid (16 mg in 1 mL) was added. The solution turned from colorless to blue immediately after the addition of 0.5 mL GNSs, indicating the formation of nanopopcorns.

#### 4.2.3. Preparing Quercetin-Loaded Nanopopcorns (QNPs)

In the third step, quercetin was adsorbed onto the surface of nanopopcorns by using EDC/NHS as a coupling agent. An EDC solution (0.1 M) was prepared by solubilizing 300 mg of EDC in 20 mL of distilled water, whereas 0.7 M NHS was prepared by dissolving 1.610 g of NHS in 20 mL of distilled water. Each solution (0.5 mL) was then added to 10 mL of the GNP solution and stirred for 1 h in the presence of 10 mg quercetin.

#### 4.2.4. Preparation of Maleylated BSA (m-BSA)

m-BSA (150 mg) was dissolved in 30 mL of 0.2 M Na_2_B_4_O_7,_ and the pH of the solution was maintained between 8 and 9 using Na_2_O_3_. To this solution, maleic anhydride was added until there was no further reduction in the solution’s pH. The solution was then dialyzed to remove unused reactants and impurities, and the obtained product was then air-dried and collected for further use.

#### 4.2.5. Preparation of Protein-Coated Nanopopcorns (PNPs)

An EDC/NHS coupling reaction was used to coat m-BSA onto the surface of quercetin-loaded nanopopcorns. Briefly, for the coupling reaction, 0.5 M EDC and NHS were added to quercetin-loaded nanopopcorns and stirred at 500 rpm for 90 min, followed by the addition of 10 mg of m-BSA under continuous stirring at room temperature. After 2 h, the solution was collected and stored at 2–4 °C in a closed vial for further use.

### 4.3. Characterization

#### 4.3.1. Particle Size (PSZ), Polydispersity (PDI), and Zeta Potential (ZEP)

The PSZ, PDI, and ZEP of the GNPs, QNPs, and PNPs were measured using a Malvern Zetasizer, UK. The PSZ and PDI were determined via dynamic light scattering (DLS), also known as photon correlation spectroscopy (PCS), while the ZEP was measured via electrophoretic light scattering. All measurements were performed in triplicate (*n* = 3) at 37 °C using quartz cuvettes.

#### 4.3.2. % Adsorption Efficiency (% AEF)

To determine the AEF of QNPs, 1 mL of the formulation was centrifuged for 10 min at 8000 rpm, and the obtained supernatant was analyzed using UV–Visible spectroscopy (Shimadzu, Japan) at 254 nm in triplicate (*n* = 3). The % AEF was calculated using Equation (1).

(1) $$\%\;Adsorption\;efficiency=\frac{Initial\;drug\;loaded-Final\;concentration}{Initial\;Concentration}\times 100$$

#### 4.3.3. In Vitro Release Study (IRS)

An in vitro release study (IRS) for QNPs and PNPs was conducted using a dialysis membrane (12,000–14,000 Da cut-off) containing 2 mL of solution and then immersed in 50 mL of phosphate buffer (pH 7.4). Aliquots were drawn at 1, 2, 4, 8, 12, and 24 h, and replenished throughout to maintain sink conditions. The collected sample was analyzed at 254 nm using UV spectroscopy (Shimadzu, Japan).

#### 4.3.4. FTIR Studies

ATR-FTIR (Perkin Elmer, Shelton, CT, USA) was utilized to identify the functional groups of auric chloride, quercetin, maleic anhydride, BSA, GNPs, QNPs, and PNPs. Small quantities of samples were deposited on the diamond crystal and analyzed using Spectrum software (version 10.7.2; Perkin Elmer, Shelton, CT, USA) between 400 cm^−1^ and 4000 cm^−1^.

#### 4.3.5. DSC Studies

The thermal behaviors of auric chloride trihydrate, quercetin, GNPs, QNPs, and PNPs were characterized using DSC (Mettler Toledo, Switzerland). A small quantity (~2 mg) of sample was kept in an aluminum pan, placed inside the DSC instrument, and analyzed between 30 and 350 °C under inert conditions.

#### 4.3.6. Morphology

To analyze the morphology of the prepared GNP, QNP, and PNPs samples, SEM (scanning electron microscopy) analysis was performed by placing the sample on the stub and drying it under a UV lamp. The specimen holder was placed in the SEM instrument (Carl Zeiss Model Supra 55, Germany), and images were obtained. TEM analysis was carried out for GNPs, QNPs, and PNPs by sonicating 1 mL of each sample for 20 min, followed by placing 0.2 µL of the sonicated sample on a copper grid and drying it under a UV lamp, and the samples were subsequently analyzed at 120 kV acceleration voltage using a TEM instrument (Tecnai T20, FEI, US). TEM images were analyzed using ImageJ software, version 1.54r. The scale bar was used for spatial calibration, and the diameter of individual nanoparticles was measured manually/using the particle-analysis function. Particle-size distribution was determined from approximately thirty individual nanoparticles selected from representative micrographs for each formulation and expressed as mean ± SD.

#### 4.3.7. Dry-Powder Preparation and Aerosolization

The PNPs formulation was converted into a dry powder suitable for pulmonary administration. The obtained dry powder was carefully collected and stored in a closed container until administration. For in vivo pulmonary delivery, an accurately weighed quantity of the dry powder was loaded into a custom-fitted tubing assembly connected to a 24-gauge syringe device (0.55 mm × 25 mm). The powder was subsequently aerosolized through the assembly and administered directly into the respiratory tract of the experimental animals. The aerosolization procedure was performed using the same device configuration for all animals to maintain consistency in administration.

#### 4.3.8. In Vitro Antimyobacterial Activity

The study was performed through CRO, where H37Ra bacterial suspension was prepared in 7H9-OADC medium (160 μL per well). The cultures were pre-incubated for 8 days at 37 °C in a humidified atmosphere containing 5% CO_2_. At baseline (0 h), samples of quercetin, GNPs, QNPs, and PNPs were introduced to the respective wells. Each well then received 20 μL of Alamar Blue reagent, and the plates were returned to the incubator. After 96 h of incubation, the microplates were removed, and fluorescence was measured using a microplate reader (BioTek Instruments, Inc., Winooski, VT, USA) at an excitation wavelength of 540 nm and an emission wavelength of 590 nm.

#### 4.3.9. Animal Studies

Animal studies were authorized by SVKM Mumbai India (ethical approval code CCSEA/IAEC/P-17/2023; approval date: 4 March 2023). Pharmacokinetic profiles of the free drug and the developed PNPs were evaluated in healthy rats. Thirty-six female Albino Wistar rats (weight range: 130–160 g) were randomly allocated into two experimental groups (*n* = 18 per group; *n* = 3 per time point). Group 1 received free quercetin as a control suspension, while group 2 was treated with the PNPs formulation at a dose equivalent to 50 mg/kg of quercetin. The treatments were administered as a dry powder for inhalation via the nasal cavity. Briefly, the dry-powder formulation was loaded into a custom-fitted tubing assembly attached to a 24-gauge syringe device (dimensions = 0.55 mm × 25 mm), and subsequently aerosolized directly into the respiratory tract. At the predefined post-administration time points (1, 2, 4, 8, 12 and 24 h), the animals (*n* = 3 per time point) were humanely sacrificed. The lungs were surgically isolated, collected, and homogenized in 1 mL saline for 10 min at 4 °C. The tissue homogenates were centrifuged at 10,000 rpm for 15 min at 4 °C. The resulting supernatant was collected, processed, and quantified for pulmonary quercetin concentration using a validated LC-MS method.

## 5. Conclusions

The protein-coated quercetin-loaded gold nanopopcorns (PNPs) demonstrated enhanced inhibitory activity against *Mycobacterium tuberculosis H37Ra* cells compared with GNPs, free quercetin, and QNPs, with an IC_50_ value of 2 μg/mL. The protein-coated quercetin-loaded gold nanopopcorns had a particle size of 153.2 ± 26.45 nm and a zeta potential of 29.3 ± 7.80 mV, with an adsorption efficiency of 87.02% attributed to the irregular shape and interaction between CTAB and quercetin. The use of EDC/NHS ensured that m-BSA was effectively coated onto the surface of nanopopcorns; PNPs showed a cumulative drug release of 79.85%, thereby providing a non-invasive drug-delivery system for pulmonary delivery of quercetin. Collectively, these findings demonstrate the potential of maleylated-BSA-coated quercetin-loaded gold nanopopcorns as a pulmonary delivery platform for quercetin. However, further studies involving macrophage uptake and intracellular antimycobacterial evaluation are required to establish the contribution of macrophage targeting and to determine the therapeutic relevance of the formulation against intracellular M. tuberculosis.

## Figures and Tables

**Table 1:** PSZ, PDI, and colloidal stability of GNP, QNP, and PNPs formulations.

Name	PSZ (nm ± S.D.)	PDI	ZEP (mV ± S.D.)
GNPs	34.42 ± 12.48	0.457	39 ± 10.98
QNPs	125.9 ± 56.89	0.361	34.8 ± 5.66
PNPs	153.2 ± 26.45	0.404	29.3 ± 7.80

**Table 2:** % Inhibition and IC50 values for GNPs, quercetin, QNPs, and PNPs.

Concentration	GNP	Quercetin Sol.	QNP	PNPs
10	12.31	52.34	53.24	56.36
1	8.97	26.42	38.16	42.37
0.1	6.34	13.28	10.06	18.63
0.01	2.34	3.57	3.64	2.08
0.001	1.54	1.06	2.33	1.06
IC_50_ (µg/mL)	>10	8	5	2

**Table 3:** In vivo pharmacokinetic parameters.

Parameter	Free Quercetin	PNPs
Cmax (µg/g)	15.60 ± 2.10	25.70 ± 2.80
Tmax (h)	2.00 ± 0.00	2.00 ± 0.00
AUC_0–24_(µg·h/g)	146.25 ± 0.00	356.35 ± 14.87
t½ (h)	7.96 ± 0.58	15.08 ± 0.14
AUC_0–∞_ (µg·h/g)	168.06 ± 11.89	545.68 ± 25.94

## Data Availability

The original contributions presented in this study are included in the article. Further inquiries can be directed to the corresponding authors.
